# Plasmonic Enhancement of Dye Sensitized Solar Cells via a Tailored Size-Distribution of Chemically Functionalized Gold Nanoparticles

**DOI:** 10.1371/journal.pone.0109836

**Published:** 2014-10-29

**Authors:** Codrin Andrei, Elena Lestini, Stephen Crosbie, Caoimhe de Frein, Thomas O'Reilly, Dominic Zerulla

**Affiliations:** 1 Plasmonics and Ultrafast NanoOptics Group, School of Physics, University College Dublin, Dublin, Ireland; 2 School of Mathematical Sciences, University College Dublin, Dublin, Ireland; Argonne National Laboratory, United States of America

## Abstract

A substantial and stable increase of the current density J_sc_ of ruthenium (Ru) dye sensitized solar cells (DSC) of up to 16.18% and of the power efficiency of up to 25.5% is demonstrated in this article via plasmonic enhancement. The key aspect of this work is the use of a tailored bimodal size distribution of functionalized gold nanoparticles (AuNPs) that have been chemically immobilized onto the mesoporous titanium dioxide (TiO_2_) layer via short, stable dithiodibutyric acid linkers. The size distribution of the AuNPs is a result of theoretical calculations that aimed at the perfection of the absorption characteristics of the complete solar cell system over a wide range of wavelengths. The functionalization of the AuNPs serves to bind them at a close but defined distance to TiO_2_-particles and additionally to chemically protect them against potential corrosion by the electrolyte. Simulations of near field (enhanced absorption) and far field (scattering) contributions have been used to tailor a complex AuNPs bimodal size distribution that had subsequently demonstrated experimentally a close to optimum improvement of the absorbance over a wide wavelength range (500–675 nm) and therefore an impressive DSC efficiency enhancement. Finally, the modified DSCs are exhibiting pronounced longevity and stable performance as confirmed via long time measurements. In summary, the presented systems show increased performance compared to non plasmonic enhanced cells with otherwise identical composition, and are demonstrating a previously unpublished longevity for iodide electrolyte/AuNPs combinations.

## Introduction

**Figure 1:**
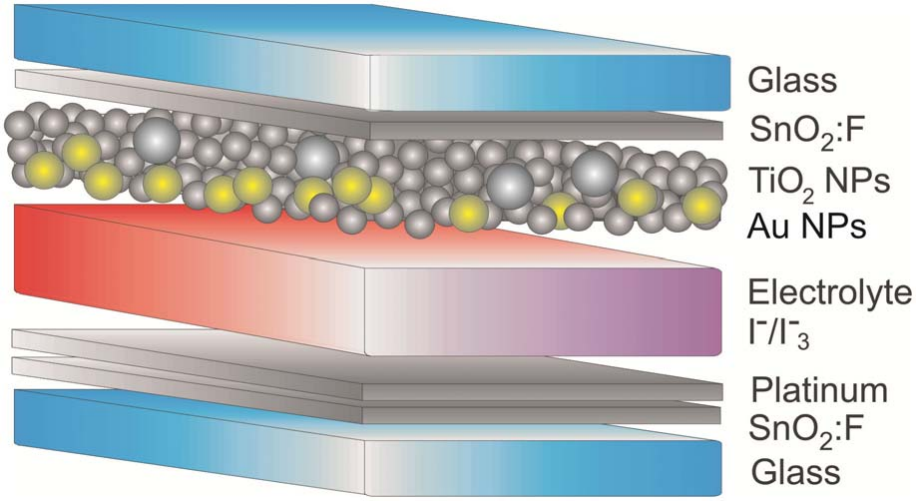
The schematic of the basic DSC design, also showing the chemically linked AuNPs plasmonic enhancement layer used herein on top of TiO_2_ particles.

Since the discovery of the photovoltaic effect in 1839 by the French scientist Alexandre Edmond Becquerel [1], researchers have been working on creating and improving photovoltaic devices that can efficiently harvest a larger portion of the 100 petawatt [2] solar energy globally provided by the sun. Invented by Michael Grätzel, the dye sensitized solar cell has become the prototype of a new generation of solar cell devices [3]. The DSC is based on a nanoparticle photoelectrode, functionalized with a light-absorbing dye, and is offering a promising alternative to silicon based solar cells, due to its advantages that include: potential lower costs of fabrication and widely available inexpensive materials [4]; the choice of both rigid and flexible designs, various shapes and transparency levels to suit domestic devices or architectural/decorative applications [4]; a con­version efficiency that does not decrease with rising environmental temperatures of up to 60°C [2]; a low level of angular dependency and thus the ability to efficiently harvest diffuse light [3]. This thin film solar cell consists of a mesoporous, semiconducting nanoparticle film onto which a monolayer of dye is adsorbed, an electrolyte, a catalytic layer and two electrodes of which one should be transparent (Figure 1).

Here, in particular, a method of functionalising the titanium dioxide film with a tailored distribution of passivated gold nanoparticles (also shown in Figure 1) is demonstrated, achieving a significant increase in DSC efficiency. This rise in efficacy is based on capturing incoming light via localized plasmonic excitations in the gold NPs. The energy of the captured light can subsequently be partially transferred to the active layer of the solar cell. Other variations to the original DSC setup are the focus of recent developments (e.g. the use of flexible electrodes [5], polymers [6], hole conductors [7], gel electrolytes [8], multi-junction devices as well as multiple exciton generation in quantum dots [4]) and show promising results for future applications. In classical photovoltaic devices that use heterojunctions composed of electron donor and acceptor elements, the charge separation and transport processes are taking place in the same material. In contrast to this scenario, in which the same material should have both high absorption potential and high carrier transport capabilities, the DSC separates the two processes of charge generation and transport. The charge generation takes place within the dye; the charge separation takes place at the interface between the dye and the semiconductor (typically TiO_2_), while the charge transport is accomplished by the semiconductor and the electrolyte. This gives more flexibility in optimising the performance of the DSC, by tuning each of its components individually, as opposed to other solar cell types. The working principle of a DSC is based on a fast electron injection (on a ps-ns scale) from an excited state of a dye into the conduction band of a wide band gap semiconductor [2]. Due to its unique properties, high abundance and therefore potentially competitive price, titanium dioxide is one of the most employed semiconductors for DSCs. The dyes play a key role in sensitising the TiO_2_ layer to the visible and near-infrared part of the solar spectrum, i.e. to enhance the wavelength acceptance of TiO_2_, as without the sensitizer the semiconductor would only respond to ultraviolet light according to its band gap [3].

The process of converting light incident on the DSC to electricity via the attached dye molecules takes place on a molecular level and is therefore, at least in this aspect, similar to the photosynthesis in nature; it is a regenerative photo electro­chemical process by way of the electrolyte. The lowest excited state of the dye must align to the conduction band edge of the semiconductor and the absorption range of the dye should cover the widest range possible, e.g. the entire visible spectrum [9] plus the near-infrared. The effective decrease of the large band gap of the semiconductor (3.2 eV for the TiO_2_ anatase phase) may be accounted for by assuming the intro­duction of ‘new’ dye surface states [3]; the range of wavelengths absorbed by a DSC is governed by this effective band gap. Natural, porphyrin­ chro­mo­­phore sensitizers may be chemically modified to yield high power conversion efficiencies of 11% [10]. Synthetic ruthenium dye complexes like the N3, N749 or N719 dyes report record efficiencies of more than 11% at present. A recent breakthrough achieved by M. Grätzel’s research group has allowed efficiencies of more than 12% to be reached in a DSC by using a cobalt based redox electrolyte in conjunction with a zinc porphyrin dye (YD2-o-C8) as sensitizer [11]. Tandem DSCs have reached efficiencies of 16% [12] and record modules with an efficiency of 8.4% have been reported by Sony Corporation[13], with 9.9% module efficiency achievable according to the same company. Before 1991, the efficiency of a DSC was less than 1% and the major step was achieved in that year, when Grätzel published the 7.1% efficiency record at that time [3]. The DSC efficiency and stability has been continuously improved over the last decade, but as recently reported by Polman and Atwater [14], there is significantly more room for improvement, suggesting that by using plasmonic enhancement in solar cells, the Shockley–Queisser limit can be surpassed in single junction cells. The concept of integrating plasmonic structures into solar cells is known for over a decade [15], [16]. Experimentally, high peak enhancements at specific wavelengths [17], and overall efficiency enhancements of 40%, 8.3%, and 8% have been demonstrated with the use of plasmonic nanoparticles for cells employing organics [18], a-Si [19], and GaAs [20], respectively.

We have already reported theoretical studies on plasmonic solar cell enhancement using nanoscale structures with multiple periodicities permitting optimisation of the cell’s response [21], whereas an experimental approach on the basis of a tailored distribution of functionalised gold-particles is investigated in the current publication. Here we have chosen gold as the plasmonic material solely for the fact that it is chemically very inert which in conjunction to the below detailed functionalization/passivation of the NPs adds to the overall stability. However, the authors wish to note that silver, especially in the visible, has superior plasmonic characteristics which may lead to even further improvements of the solar cells’ efficiency. Chemical inertness is less an issue if an all-solid-state solar cell concept is chosen.

The efficiency of a DSC based on the Ru N3 has been unmatched since its invention by Grätzel in 1993, until 1997 when he developed the Ru N749 dye. Ru N719 has played a major role in advancing the DSC technology as it has high V_OC_ and I_SC_ [22].

The preparation of the DSC photo-anode includes the sintering of the applied TiO_2_ film, required for efficiently removing potential organic binders and surfactants used in the TiO_2_ preparation and importantly for establishing good electrical interfaces between adjacent TiO_2_ nanoparticles in the mesoporous layer, as well as between the TiO_2_ nanoparticles and the transparent conductive oxide (TCO) layer. The TiO_2_ sintering temperature of 450°C was reported to produce good electrical contacts between the nanoparticles and the TCO (e.g. SnO_2_:F), but also between the nanoparticles that form the scaffold [23]. The sintering process and its associated processes (e.g. the Sn diffusion [24]) play a crucial role in the DSC performance, as a failure in achieving good electrical contacts will be automatically translated into low cell per­formance. To increase the efficiency of a DSC, the short circuit current (I_SC_), the open-circuit voltage (V_OC_) and the fill factor (FF) need to be improved. The I_SC_ can be increased for instance by engineering dyes with near-infrared absorption capabilities; the V_OC_ enhancement requires the optimization of the band edge and a decrease of recombination processes; the FF improvement requires to decrease the losses at the dye regeneration and to improve the electron transport. A thick layer of TiO_2_ (>25 µm) translates into a larger effective surface area for the dye adsorption, but it will also increase the effective path length for the electrons to travel to the electrode, and thus the average resistivity of the system, and additionally might create a higher recombination rate that lowers the DSC’s quantum efficiency (although, unlike e.g. polymer cells, DSC’s don’t suffer strongly from recombination losses). The solution is to decrease the thickness of the TiO_2_ mesoporous layer (down to 10 µm or less) in order to decrease the path length for the electrons to travel. By this, the mean electron diffusion length in a DSC, measured to be in the range of 10–20 µm [25], is set optimally to achieve a high DSC efficiency. Although decreasing the TiO_2_ thickness reduces the dark current [26], it also reduces the dye adsorption active area and the photocurrent. This can be overcome by using high extinction coefficient dyes, scattering particles (e.g. ∼190 nm TiO_2_ crystals) to increase the light path, plasmonic enhancement or a combination of these, as demonstrated in this article.

The use of plasmonic effects for light absorption and efficiency enhancement in Si-based solar cells has been previously reported [27]–[33], and for DSCs using a periodic nanodome pattern has been recently reported by Ding et al. in 2011[34]. Other examples with promising results include light trapping nanopatterns, nano­structured plasmonic solar cells and light trapping in ultrathin plasmonic solar cells [35]–[38].

Here, a different approach with significant plasmonic enhancement and its associated improved light absorption and therefore DSC efficiency increase is reported. The here presented research is focusing on functionalised gold nanoparticles with a synthesised, optimally tailored size distribution. Additionally, their func­tionalization protects the nanoparticles against the electrolytic corrosion leading to stable, efficiency improved DSCs.

## Experimental Procedure

The DSCs electrodes have been prepared on aluminoborosilicate (AlBSi) glass slides (Schott AG, n = 1.516, 2 cm x 2 cm x 1.1 mm) that have been coated with a 700 nm thick SnO_2_:F layer (10 Ω sq^−1^) by spray pyrolysis at 600°C.

The photoanodes have been fabricated by depositing TiO_2_ (∼2.8 µg) nanoparticle paste (Solaronix SA) on each FTO glass slide (TiO_2_ area 1.69 cm^2^ for each electrode) via the tape casting technique [39]. Prior to the deposition process, the glass slides were subject to a sonication treatment for 30 minutes at room temperature, subsequently rinsed with distilled water and ethanol (Sigma-Aldrich, 99.8%), and then allowed to dry in a clean sealed container. The TiO_2_ paste used herein was a compound of 11% wt. nanocrystalline anatase TiO_2_ mixed with larger, optically dispersing, anatase particles. A FEI Quanta 3D dual beam scanning electron microscope equipped with a highly spatially resolving electron field emission gun, a Gallium ion gun and an EDAX Trident XM4 energy dispersive X-ray detector, was employed for imaging and depth profiling of the solar cell’s active layer structure, providing a high spatial resolution of down to 1.5 nm. An average diameter of 20 nm for the TiO_2_ nanoparticles was measured herein via SEM, while an average diameter of 190 nm was determined with the same technique for the optically dispersing TiO_2_ particles (Figure S1).

Subsequent to the TiO_2_ paste deposition, the photoanodes have been sintered by programming a temperature controllable oven to ramp-up from room temperature to 450°C, under normal atmospheric conditions, with a heating rate of 20°C min^−1^. The 450°C limit was kept constant for 20 minutes, followed by a smooth cooling process. The photoanodes have been subsequently post-treated with a TiCl_4_ solution (50 mM, Sigma-Aldrich, 99.0%) at 60°C for 20 minutes. Then, the electrodes were thoroughly rinsed with distilled water and re-sintered at 450°C for 20 minutes.

The deposition of AuNPs on the TiO_2_ surface was carried out using dithiodibutyric acid (50 mM, *Sigma Aldrich,* 95%) ligand solution in methanol (*Sigma Aldrich,* 99.8%). The TiO_2_ elec­trodes were fabricated as described above, allowed to cool down after the TiCl_4_ post-treatment to 100°C, and then immersed into the ligand solution for 2 hours at room temperature. The electrodes were then thoroughly rinsed with methanol, subsequently heated for 10 minutes at 80°C and cooled down to room temperature. The ligand-functionalized TiO_2_ films were inserted into a gold nanoparticle solution (a mix of self-synthesized particles with a tailored size distribution and commercially available (spherical) AuNPs *Nanopartz,* wt 1.75%, molar extinction coefficient 2.10×10^9^ M^−1^ cm^−1^) and left for 18 hours in the dark, at room temperature, in a sealed glass container. The size distribution of the gold nanoparticles can be tailored to match perfectly the necessary wavelength dependent enhancement of the DSC in the visible and NIR regions. One of the chosen nanoparticle size distributions is centered on a diameter of 100 nm as shown in the above SEM images.

The electrodes were subsequently rinsed with deionized water, dipped triply in separate beakers containing methanol, rinsed once with acetone (*Sigma Aldrich*, 99.8%) and then dipped once again in methanol. The electrodes were finally heated for 10 minutes at 70°C and then it followed the ruthenium adsorption. State of the art ruthenium dyes N719, N749 and N3 solutions (0.2 mM) have been made in an ethanol solution. Chenodeoxycholic acid (3α,7α-Dihydroxy-5β-cholanic acid) solution in ethanol (20 mM) has been added as a co-adsorbent to the Ru dyes solutions. Subsequent to the AuNPs functionalisation, the TiO_2_ electrodes were cooled to 60°C, and immersed into one of the three ruthenium dye solutions mentioned above, for 24 hours (sealed environment, in dark, at room temperature) then thoroughly rinsed with absolute alcohol (*Fluka*, 99.8%) and finally assembled into DSCs.

The counter electrodes have been fabricated by using same parameters AlBSi glass slides and FTO as described above. A thin layer of hexa­chloroplatinic acid (5 mM H_2_PtCl_6_ in iso­pro­panol) was deposited on the FTO and was followed by a 15 minutes sintering process at 450°C, with an average ramp-up rate of 20°C min^−1^. The electrolyte was iodide/tri-iodide (50 mM) redox couple, with pyridine derivative as additive and with propionitrile as solvent.

The XPS measurements were performed on a VG Scientific Microlab 310D high-resolution photo­electron spectrometer, using its Mg Kα X-ray source (hν  = 1253.6 eV). A base pressure of 1×10^−9^ mbar was recorded for all of the measurements. The electron kinetic energies were measured by a hemispherical analyzer, normal to the samples surface. Spectral decomposition was performed using the dedicated program suite Unifit 2012.

The Raman spectra were measured using a Holospec f/1.8 Holographic Imaging Spectro­graph (Kaiser), and the laser excitation of 532 nm (Laser Quantum - Torus, CW stabilized single frequency Nd:YAG laser) at normal incidence on the samples was detected with an Andor Newton EMCCD.

## Results and Discussion

The goal of “establishing a plasmonic nano­particle enhanced solar cell with improved efficiency and long lifetime” requires an initial, thorough characterisation of the TiO_2_, AuNPs and TiO_2_-AuNPs interfaces using XPS, SEM, FIB and additionally quantification of the Ru-based DSCs efficiency improvement achieved.

**Figure 2:**
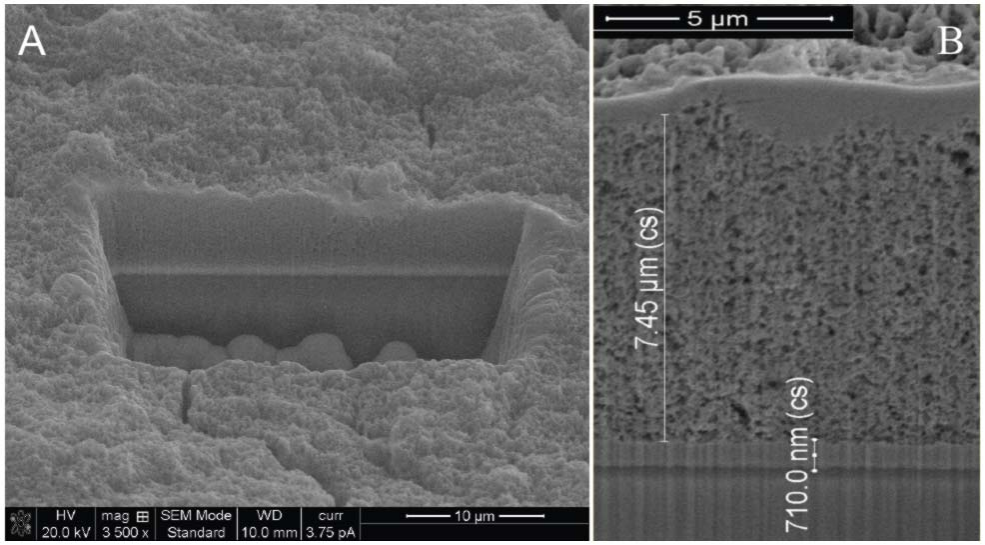
SEM image subsequent to focused ion beam milling to evaluate the absolute thickness of the TiO_2_ layer; magnification 3500*x* and scale bar 10 µm (A). SEM confirming the TiO_2_ average thickness of 7.45 µm, magnification 12500*x* and scale bar 5 µm (**B**).

The TiO_2_ layer thickness was kept just below 10 µm (Figure 2B) which translates into an efficient electron collection [25]. In order to evaluate the precise absolute thickness of the TiO_2_ scaffold subsequent to the sintering, a cuboid with the dimensions of 20 µm x 30 µm x 20 µm was removed from the TiO_2_/SnO_2_/AlBSi heterojunction, using focused ion beam milling (FIB) techniques (Figure 2A).

**Figure 3:**
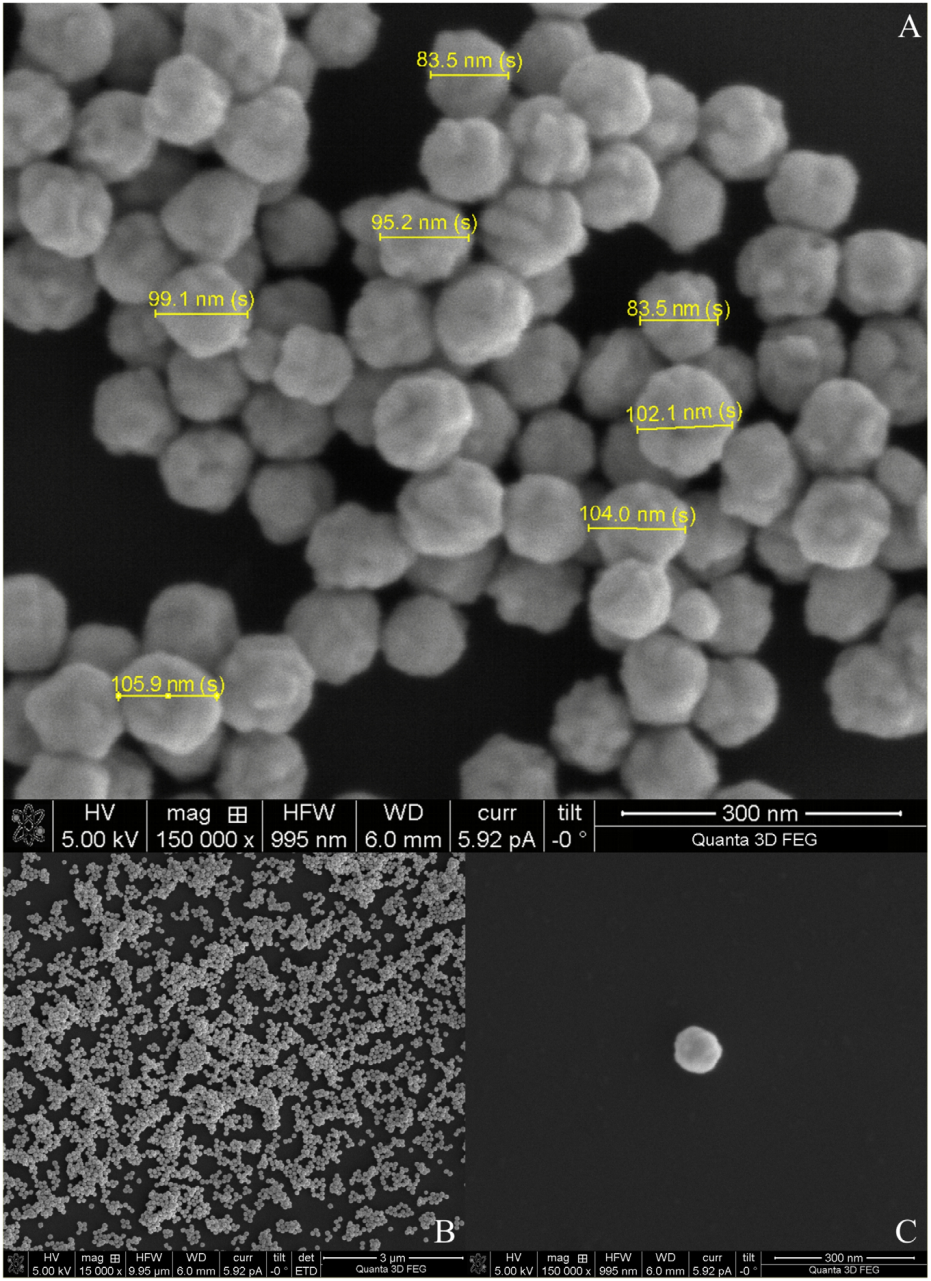
SEM image of the AuNPs immobilized on Si (A); SEM image showing the AuNPs dispersed on Si wafer (B); SEM image of a single AuNP; magnification 150,000x and scale bar 300 nm (C).

Subsequent to the FIB milling, SEM measurements revealed an average thickness of the TiO_2_ layer of ∼7.45 µm (Figure 2B). Initially, for imaging purposes only, the AuNPs were immobilized on flat Si wafers and imaged using the same FIB/SEM system (Figure 3).

Imaging the NPs on Si instead of on mesoporous TiO_2_ results in a higher quality of the quantitative results, in particular the here important size distribution of AuNPs. By immobilising the AuNPs on a Si wafer and subsequent imaging, it was confirmed that the necessary size distribution of the larger AuNP species is within the bimodal size distribution, as requested by the below simulations. Closer inspection of the images reveals also that the NPs that have been purpose made for the here discussed DSC’s are not perfectly spherical. As a result of the colloidal synthesis procedure employed, the NPs exhibit protrusions and look partly polyhedral in their appearance, clearly due to different crystallographic domains which are joined in these particles (Figure 3A, C).

The plasmonic response of the particles will be slightly altered by their mildly differing shape as can be demonstrated using numerical simulations including Discrete Dipole Approximation (DDA) suitable for arbitrarily shape particles [40]–[42]. However, in the light that we are investigating a large ensemble of NPs, the absence of an analytical function to describe their precise shape, and the fact that each particle is slightly different than its neighbour we believe it is reasonable to keep our model simple in this regard in assuming a spherical shape and to limit our investigations at this time to the size distribution of interest within this framework.

**Figure 4:**
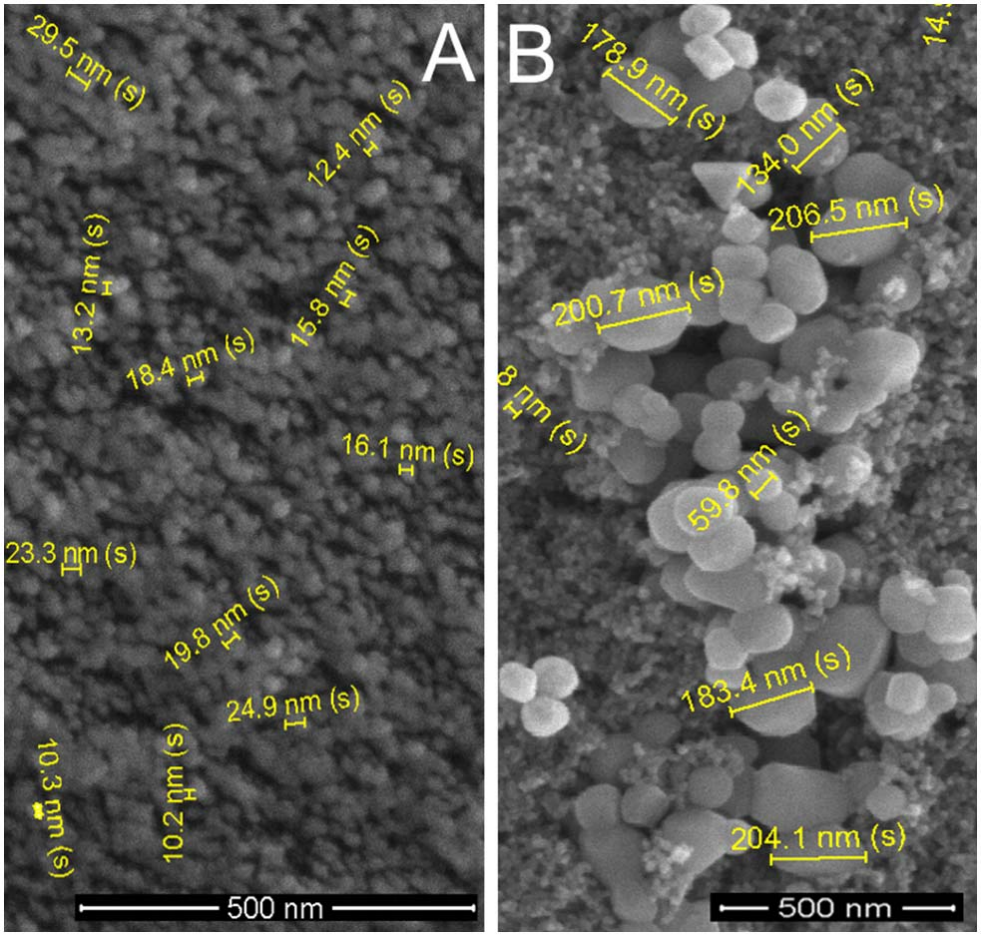
SEM image of the TiO_2_ nanoparticles (Ø ∼20 nm), with optically dispersing TiO_2_ particles (Ø ∼190 nm) and functionalized with AuNPs; magnification 50,000*x* and scale bar 500 nm.

In a next step, the functionalised gold particles using dithiodibutyric acid linker are shown together with the two different types of TiO_2_ nanoparticles (small NP Ø ∼20 nm and large NPs Ø ∼190 nm) located on top of a sintered TiO_2_ mesoporous layer (Figure 4). As expected, the AuNPs are attached to both the 20 nm diameter TiO_2_, as well as to the larger (Ø ∼190 nm) optically dispersing anatase TiO_2_.

The AuNPs can be distinguished from the larger TiO_2_ particles not only by their lower diameter (Ø ∼100 nm), but also by their increased secondary electron scattering yield compared to the TiO_2_ (Figure 4). The SEM analysis shows a good dispersion of AuNPs on the TiO_2_ surface, which is an important feature required to obtain the optimal particle number to performance ratio. Functionalising the TiO_2_ layer with the linker and only subsequently adding the AuNPs to the system, allowed the formation of a (sub)-monolayer of AuNPs (Figure 4 B), whereas by firstly attaching the linker to the AuNPs would have caused unwanted aggregation of the AuNPs. A good dispersion of AuNPs has been achieved by tuning the concentration of the dithiodibutyric acid linker to 50 mM. From the SEM analysis, comparing the results shown in Figure 3 (before the TiO_2_ functionalisation with AuNPs) and Figure 4 (after the functionalization) it can be concluded that the chemical preparation (including the 10 minutes sintering at 70°C) had no effect on the AuNPs diameter which is a crucial property for the here desired plasmonic effects. Simulations of the AuNPs Mie scattering, based on a precisely tailored NP distribution used to enhance the dye absorbance, are shown below.

**Figure 5:**
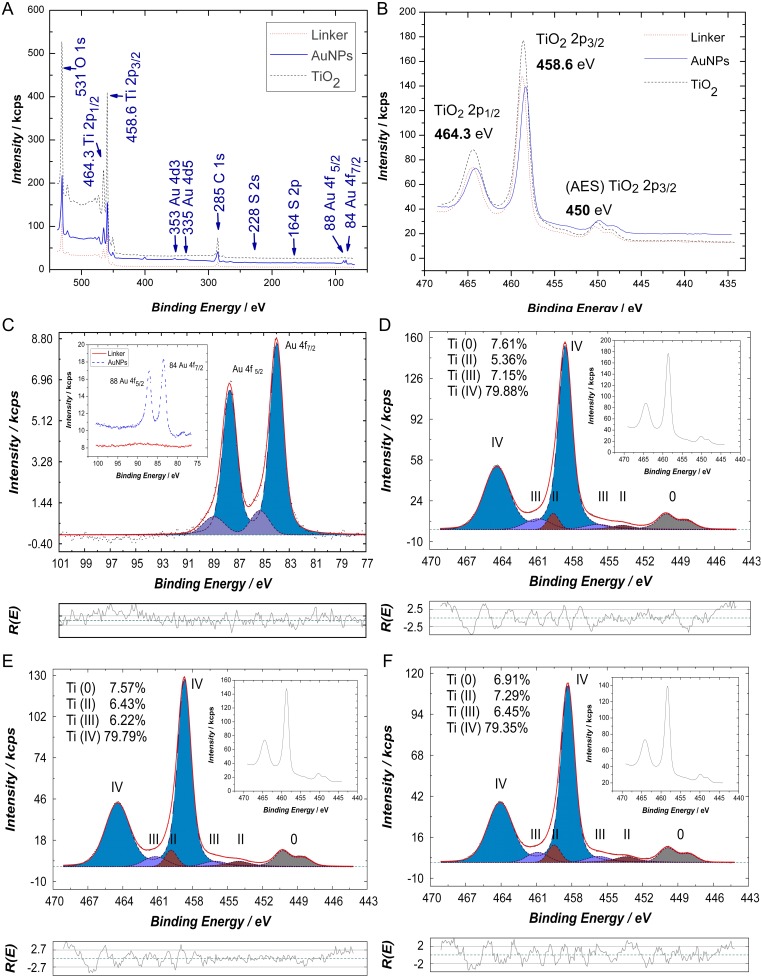
The XPS analysis confirming the TiO_2_-S and the S-AuNPs bonding. (**A**) The survey; (**B**) The effects of the AuNPs functionalization on the TiO_2_ (shifts of the Ti 2p peaks); **(C)** Confirmation of the AuNPs presence (Au 4f) and the peak fitting; (**D**) The XPS peak fitting of Ti 2p for pure TiO_2_ samples; (**E**) The XPS peak fitting of Ti 2p for samples with TiO_2_ and dithiodibutyric acid linker; (**F**) The XPS peak fitting of Ti 2p for samples with AuNPs attached to the TiO_2_ via the linker.

Further confirmation of the AuNPs bonding to the TiO_2_ surface via short linkers as shown in the above SEM images, was sought through a complementary technique using detailed XPS spectra confirming the bonding of AuNPs-TiO_2_ and to extract further information regarding the chemical state of the system (Figure 5). The survey spectra were taken from 70 eV to 550 eV (Figure 5A). The linker ([-S(CH_2_)_3_CO_2_H]_2_) presence was confirmed by identifying sulphur species as shown in Figure 5A, at 164 eV for S 2p and even the weaker S 2s peak was found at 228 eV. The XPS spectra in the 75 eV–100 eV region were carried out to confirm the presence of AuNPs, and the same dwell time (500 ms) and step size (0.1 eV) as for the S peaks have been used. As known from previous publications, upon contact with the Au NPs the dithiodibutyric acid linker (a dithiol) will break into two alkanethiols whose sulfur(s) will bind to the Au and whose carboxylic groups will bind to the titania, thus immobilizing the AuNPs at a short but defined distance to the TiO_2_ particles [43], [44]. Figure 5C shows the two peaks corresponding to Au 4f7/2 (84.0 eV) and Au 4f5/2 (88.0 eV) and confirms the presence of Au in the metallic state on the TiO_2_ surface. The XPS spectra in the 435 eV–470 eV range were carried out to reveal the Ti peaks (step size of 0.1 eV). The analysis of the doublet (Figure 5B), corresponding to Ti 2p3/2 and Ti 2p1/2, reveals the following: when samples with no linker and no AuNPs were scanned, the two peaks were found at 458.6 eV and 464.3 eV respectively. These values correspond to the anatase phase of TiO_2_ that has binding energy values of 458.66 eV for Ti 2p3/2 and 464.32 eV for Ti 2p1/2 [45]. Furthermore, a shift of 0.1 eV compared to the above mentioned values was detected, for both Ti peaks, when investigating TiO_2_ bound to the linker. A shift of 0.3 eV was recorded for both Ti peaks when samples containing also the AuNPs linked to the TiO_2_ were investigated. A shift in the same direction, but larger (0.6 eV), has been reported by Liu et al. when investigating 1 nm AuNPs deposited directly onto TiO_2_ [46]. A precise chemical analysis was achieved by peak fitting the XPS Ti 2p peaks for samples with just TiO_2_ (Figure 5D), for samples with TiO_2_ and the linker, and for samples with AuNPs linked to the TiO_2_ surface. The majority of the Ti2p signal stems as expected from Ti(IV). However, the nanocrystals seem to contain intrinsically also a small fraction of less oxidized Titanium species (which we tentatively assign as Ti(II)) which indicates a non stoichiometric form or mixed crystal character. A detailed investigation shows that when chemically modifying the titania layer with the linker and subsequently with the AuNPs, the low Ti(II) signal increased from 5.36% (when no linker was used) to 6.43% in the linker’s presence, and furthermore to 7.29% when AuNPs were added to the system. Also Ti(IV) was slightly decreased from 79.88% (when no AuNPs were used) to 79.79% in the linker’s presence on TiO_2_, and furthermore to 79.35% when AuNPs were chemically attached. This decrease can be associated with the linker coverage of the titania surface after the functionalization, and together with the quantified sulphur species analysed on the active layer surface, it concludes via this XPS analysis that the linker has chemically connected the AuNPs with the TiO_2_ interface.

**Figure 6:**
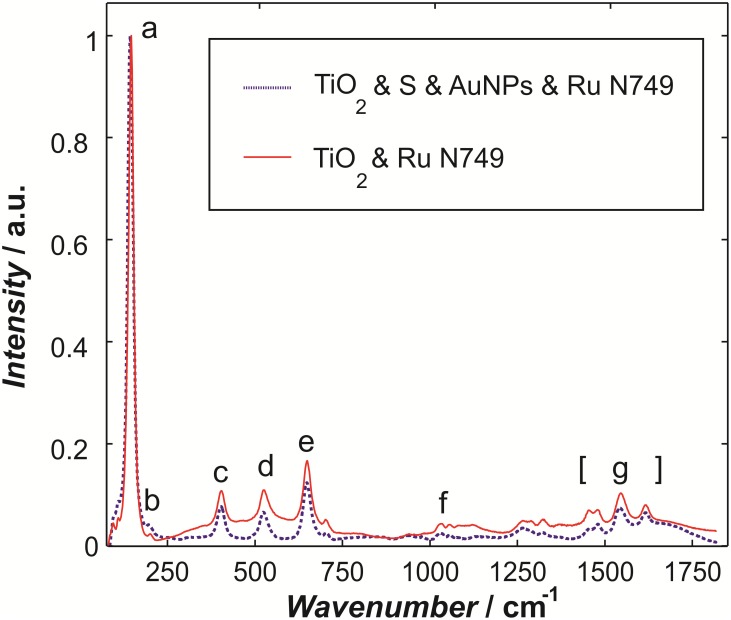
Raman spectra of TiO_2_ samples with Ru N749, and of TiO_2_ samples with AuNPs plus Ru N749.

In order to further investigate the effect of functionalization TiO_2_ with AuNPs and its potential effect on suppressing the ad­sorption of Ru molecules on the TiO_2_ surface, Raman scattering was additionally used for samples of Ru N749 sensitized TiO_2_ films with and without the AuNPs. The Raman spectra are shown in Figure 6 and the identified modes are further discussed. However, this method is non-quantitative and it only indicates that the AuNPs attach to the linker subsequent to the functionalisation of TiO_2_, without affecting the adsorption of Ru to the titania layer. Future Raman studies will examine primarily the signatures of S-Au and C-S stretches for quantifying the linker presence and functions in the present configuration.

The measured Raman modes (Table 1) can be assigned to the Raman spectra of anatase single crystals as reported in literature [47]: ∼149 cm^−1^ (E_g_), 200 cm^−1^ (E_g_), 408 cm^−1^ (B_1g_), 526 cm^−1^ (B_1g_) and 650 cm^−1^ (E_g_). The mode at 1033 cm^−1^ is the ring mode of Ru N749, while the modes in the 1400–1650 cm^−1^ regime are the terpyridine ligands of Ru N749 [48]. These peaks confirm the presence of the Ru N749 in both samples despite the addition of the gold nanoparticles.

**Table 1:** Raman modes and their assignment to either TiO_2_ or Ru N749.

Letter on graph	Mode	Wavenumber [cm^−1^]	Assignment
A	E_g_	149	Anatase TiO_2_ [47]
B	E_g_	200	Anatase TiO_2_ [47]
C	B_1g_	408	Anatase TiO_2_ [47]
D	B_1g_	526	Anatase TiO_2_ [47]
E	E_g_	650	Anatase TiO_2_ [47]
f	Ring	1033	Ru N749 [48]
g	Terpyridine ligands	1400–1650	Ru N749 [48]

The detection of modes ‘f’ and ‘g’ for the AuNPs functionalized samples indicates the presence of ruthenium molecules.

By confirming the attachment of AuNPs to the TiO_2_ nanoparticles by both SEM and XPS techniques, and by confirming the presence of Ru N749 on the titania layer subsequent to AuNPs attachment by Raman spectroscopy, the chemical AuNPs bonding method developed herein was characterised.

Prior to the testing of fully assembled DSCs including the functionalized plasmonic particles, we would like to focus onto the model behind the AuNPs’ size distribution. Firstly, the authors wish to critically remark that neither plasmonic absorption alone nor optical absorption of the fully assembled cells are good indicators for solar cell performance because it is not immediately clear if the absorption leads to enhanced IPCE of the cell or the absorbed light is thermalized instead. In order to forecast the total performance of solar cells including embedded nanoscale features sophisticated modelling of the complete field distributions (of the spatially non-symmetric environment) and explicit models for the energy transfer between metallic NPs and the semiconductor structures are necessary. We have shown recently, that the plasmonic field distribution can be tailored with the help of a complex topography in such a way that the field can be partially moved away from the metal and made stronger in the active layer. This leads to decreased thermalization of the plasmon in the metal and results in an overall improvement of solar cells efficiency. However, we have deliberately restricted the scope of this paper to spherical NPs which don’t have the extra degrees of design parameters necessary for such an approach. Their size, modelled here in an effective medium environment, determines directly their absorption characteristics which need to match the incoming light and the absorption deficiencies of the solar cells.

In order to see the overall effect on the IPCE, we have first determined the size distribution based on the simulations (extinction, absorption and scattering) with an emphasis on the harvestable region of the sensitizer dye, and then finally experimentally measured the influence of the added gold NPs on the IPCE of the cells instead, as detailed below.

**Figure 7:**
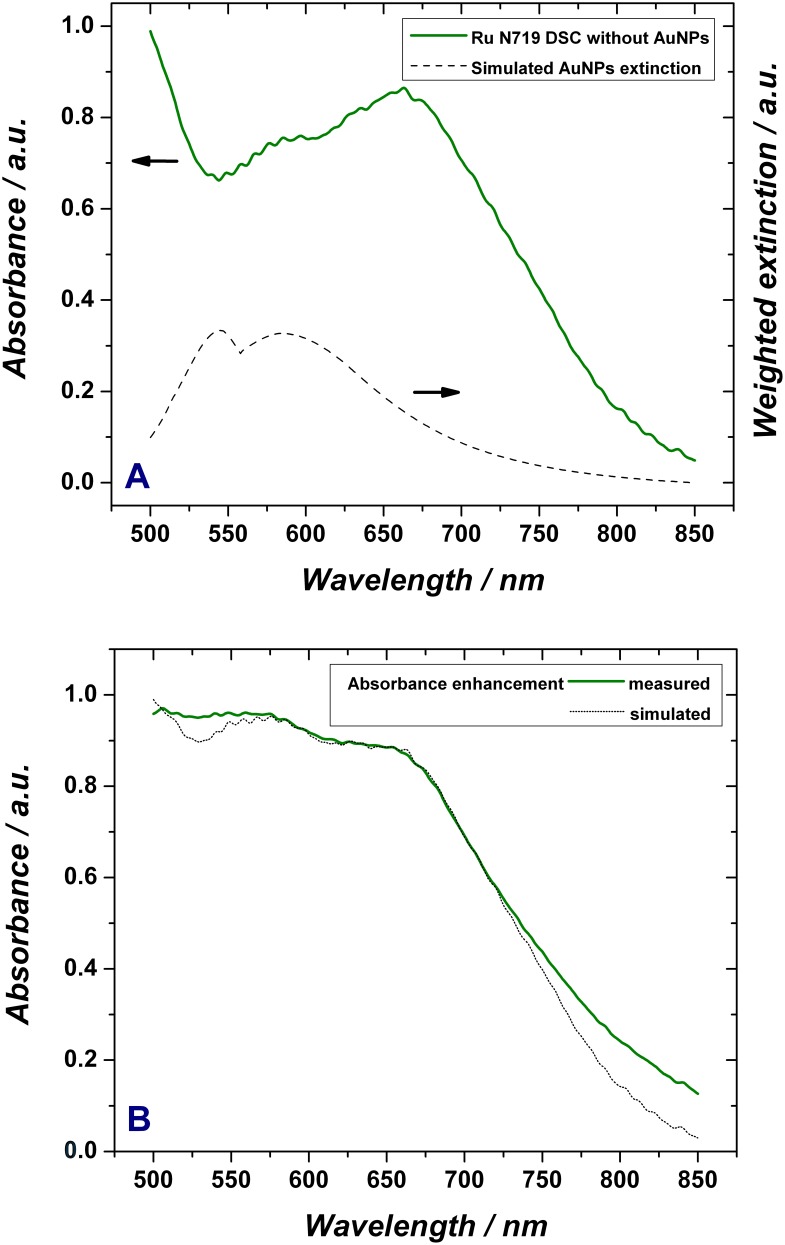
The absorbance as a function of wavelength for Ru N719 without AuNPs and the simulated AuNPs extinction (A); Measured and simulated absorbance enhancement of Ru N719 DSCs when using tailored AuNPs (B).

As a next step, absorbance spectra of TiO_2_ electrodes sensitized with the best performing ruthenium dyes have been made. The absorbance of the system sensitized with the Ru N719 dye, as a function of the wavelength is shown in Figure 7A (green curve). It clearly shows good absorption for wavelength around 500 nm but the performance drops considerably for larger wavelengths with a local minimum close to 540 nm, a slight increase towards 660 nm, and then a long decrease towards the NIR.

Ideally, the absorption should be kept close to 1 for all wavelengths which contribute to the photon generated current. In order to improve the dyes effective absorption in the visible we have added AuNPs of a below described bimodal distribution whose extinction (combined ab­sorption and scattering) peaks close to 545 nm (corresponding to particles which are smaller than 40 nm and whose absorption characteristics are according to Mie theory independent of their actual size) and peaks close to 585 nm with a long tail towards the NIR (corresponding to a tailored size distribution of AuNPs from 70 nm to 110 nm) as also shown in Fig. 7A (dashed curve). Gold nanoparticles increase the absorption of light in two ways: they scatter the light (similar to the TiO_2_ particles with diameters of ∼200 nm) into the far field and they absorb light whose energy is then available for dye molecules in an evanescent (exponentially decaying) near field. For the former, due to the position of the Au nanoparticles which is mainly towards the back of the mesoporous TiO_2_ system, the effective path length is strongly increased when backscattering appears (90 to 270 degrees). For the latter, the near field enhancement, the mechanism is entirely different. The absorbed photons are exciting a localized plasmon which in turn has a certain lifetime which in the absence of radiation damping would characterise the thermalization process. However, in the presence of strongly absorbing dyes, this lifetime is dominated by the radiation damping channel which transfers the energy from the plasmons to the sensitiser molecules.

It is important to note that the different cross sections for both processes describe the probability for the occurrence of the respective phenomenon. This should not be confused with the probability of each process to finally successfully excite a dye. For the scattering, this results in an increased path length and therefore an increased probability for the absorption somewhere in the active layer. For the localized plasmon, an increased lifetime (compared to free space light) raises the probability for excitation of dyes which are within the evanescent field which is exponentially decaying perpen­dicular to the Au particle surface on typical length scales of ∼100 nm. The authors wish to remark that the ratio of near-field absorption to far-field scattering is, for the discussed spherical Au NPs in a given effective medium modelled environment, basically a function of the NP diameter. However, as size is the only tuning parameter in this investigation, we cannot tailor for wavelengths dependent absorption and the near-field/far-field ratio independently. This would require more complexly shaped NPs.

Interestingly, using the Maxwell Garnett effective medium approximation for the electrolyte filled TiO_2_-system (n_eff_ = 1.74), both cross sections are of the same order of magnitude. More precisely, smaller particles (i.e. <40 nm) have a stronger absorption than scattering (but of similar magnitude), larger particles (>80 nm) have more absorption up to λ = 545 nm but more scattering beyond this value. Furthermore, the scattering becomes more dominant when the particles get larger. The superposition results of the extinction (scattering plus absorption) of an AuNP ensemble for the desired wavelengths range using an optimised NP size distribution and taking all above described effects into account is depicted in Fig. 7A.

It is clearly visible that the simulated extinction of the AuNPs, based on the two above mechanisms, was tailored to complement the Ru N719 absorbance in the area of 500–675 nm. In fact, although the AuNPs are being located only in the lower sections of the active layer for manufacturing reasons, both contributions (scattering & absorption into localised plasmon modes which concentrate and confine the light close to the NP’s where it can be efficiently transferred to dyes in their close vicinity) clearly improve the absorption of the solar cell and are also leading to an improved current density as shown further below. The weighted AuNPs extinction simulated in this model has taken into account the fact that the AuNPs local environment also varies strongly (the individual AuNP environments will vary primarily depending on the binding position relative to the TiO_2_ mesoporous layer which is a high refractive index material and therefore is dominating the local refractive index perceived by the NPs) and therefore results in a broad distribution of effective optical constants, which as a result broadens additionally the plasmonic resonance.

For systems needing a larger tuning range of the plasmon resonances than the here presented DSCs rod shaped particles are recommendable where the resonances depend on the aspect ratio of the long to short axis instead of the absolute diameter. However, the simulations demonstrate that the here presented bimodal AuNP size distribution with two extinction peaks at 545 nm and 585 nm (Figure 7A) is suitable to complement the dye absorbance enhancement in the range of 500–700 nm. Figure 7B shows the simulated absorbance of a plasmonically enhanced cell (doted black curve), achieved when incorporating the above described AuNPs size distribution. This demonstrates that by using this plasmonic enhancement the absorbance can be kept between 88% and 98% over the entire wavelength range of 500–675 nm. Furthermore, Figure 7B shows the experimental measurement of the absorbance of Ru N719 with size tailored AuNPs (green curve) which was recorded otherwise under the same conditions as the spectrum in Fig. 7A and strongly confirms the validity of the models applied to the previous simulations when using the calculated optimal NP size distribution with its associated refractive indices. Moreover, the experimental data exceeds the simulations slightly in quality which is because of the fact that the size distribution does not have to follow a functional description (as used in the simulations to represent the desired distribution) but can be mixed to complement the deficiencies of the selected dye at will. This is further illustrated by the next set of measurements which aimed to additionally validate the plasmonic enhancement by a tailored AuNPs distribution by measuring the electrical performance of standard DSCs compared to the plasmonic enhanced ones developed herein. Photocurrent and voltage measurements under simulated 1 sun AM 1.5 irradiance were quantitatively recorded with a Keithley 6514 electrometer. DSCs based only on the three ruthenium dyes were labelled N3, N749 and N719, while the DSC incorporating additionally AuNPs were labelled N3NPs, N749NPs and N719NPs, respectively.

**Figure 8:**
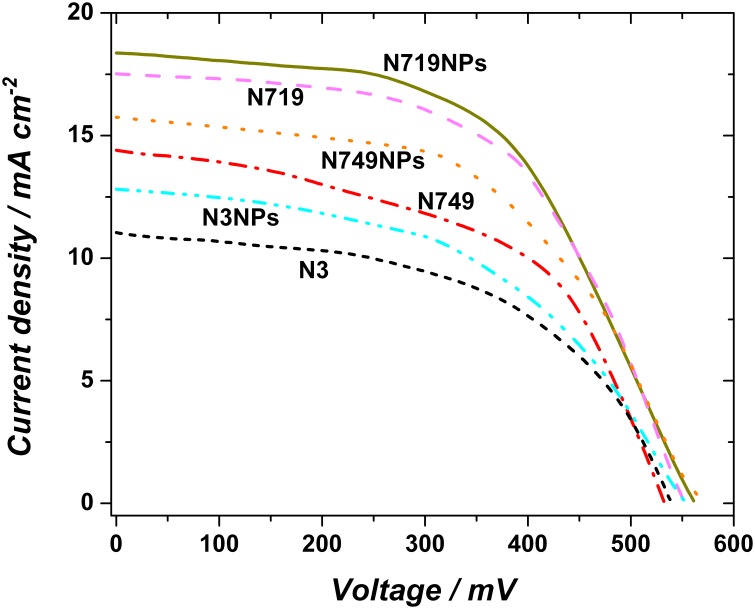
Comparison of the J-V curves of DSCs based on N719, N749, N3 and DSCs based on dyes plus AuNPs (N719NPs, N749NPs, N3NPs).

As it can be seen from Figure 8, when the AuNPs are chemically bound to the TiO_2_ as investigated herein, the DSCs show a significant increase in current density. The increase in J_SC_ for N3NPs (compared to N3) is the highest: 16.18%, followed by a measured increase of 9.29% for the N749NPs and 4.67% for N719NPs. This increase is attributed to the chemically immobilised AuNPs, which enhance the solar harvesting via particle size and wavelengths dependant far field scattering and plasmonic near field absorption with subsequent energy coupling to the sensitizer dyes. However, the dye needs to be able to efficiently convert photons for these wavelengths, and therefore, not all the ruthenium dyes will show the same increase in efficiency but the increase will vary with the dyes as determined here. It is also confirmed, the better a dye absorbs across the entire wavelengths range the less it benefits from the enhanced absorption caused by the AuNPs. By using a tailored plasmonic enhancement approach, lower efficiency dyes (e.g. N3) have a higher increase in J_SC_ compared to already high efficiency dyes (e.g. N719) as demonstrated here. Through the present approach based on the AuNPs chemical functionalization, a plasmonic near-field coupling feature can be depicted and used to increase the DSC efficiency. As mentioned before, the AuNPs also act as light scatterers, enabling a longer optical path-length. In order to increase the optical path lengths within the mesoporous TiO_2_ structure consisting of average TiO_2_ nanoparticle of Ø ∼20 nm, as mentioned before, additionally larger TiO_2_ nanoparticles of Ø ∼190 nm have been incorporated in the mesoporous film. Therefore, the measured increase in (quantum) efficiency can be mostly attributed to the plasmonic enhancement produced by chemically binding this tailored distribution of AuNPs to the TiO_2_ through the described method. The full results are shown in Table 2, and they include the increase in J_SC_ (increase of up to 16.18%), V_OC_ (increase of up to 6.8%) and P_max_ (increase of up to 25.5%).

**Table 2:** DSC performance parameters, showing the overall increase (in %) achieved herein predominantly for the J_SC_, when using the proposed experimental approach of plasmonic enhancement.

Dye	J_SC_ [mA cm^−2^]	V_OC_ [mV]	FF	Efficiency	J_SC_ increase [%]	V_OC_ increase [%]	Pmax increase [%]
N3	11.0	538	0.47	2.78	-	-	-
N3NPs	12.78	552	0.49	3.46	16.18	2.6	**25.5**
N749	14.41	532	0.51	3.91	-	-	**-**
N749NPs	15.75	568	0.52	4.65	9.29	6.8	**18.9**
N719	17.53	550	0.54	5.21	-	-	**-**
N719NPs	18.35	561	0.58	5.97	4.67	2.0	**14.6**

Wang et al. have recently reported an enhanced photocatalytic activity of Ti when using AuNPs of smaller diameters (in the order of nm) [49]. Other significant review papers on plasmonic photocatalysts have previously reported on the preparation and application of plasmonic enhancement based on noble metals[50]. The further DSC enhancement achieved herein when using a tailored size distribution of AuNPs, was confirmed both by simulations and experiments. The dye absorbance and DSC efficiency enhancement are attributed to the plasmonic enhancement achieved by calculating and validating the optimum size distribution of AuNPs used herein to functionalize the titania layer prior to ruthenium dye adsorption.

## Conclusion

Titanium dioxide films linked with a tailored, complex size distribution of gold nanoparticles via chemical functionalization, and sensitized with ruthenium complexes, have been assembled into dye sensitized solar cells and tested. Simulations of the optimum distribution of AuNPs in the context of a model for a distribution of effective refractive indices due to variations of the AuNPs local environments have indicated that for the here described systems, using a bimodal size distribution is ideally suited to optimize the absorption, and as verified the efficiency of the DSCs over the entire visible wavelength range. A chemical self-assembly procedure for linking the AuNPs to the mesoporous active layer was introduced, followed by a full characterization and testing of the system. X-ray photoelectron spectroscopy has provided a detailed insight into the chemical binding of the AuNPs to the TiO_2_ via the chosen short dithiodibutyric acid linker, and in addition the spatial distribution and configuration of this nanoassembly was investigated by using scanning electron microscopy, and focussed ion beam in conjunction with scanning electron microscopy. Furthermore J-V measurements demonstrate that this model accounts for an appreciable increase of the current density J_SC_ and thus of the solar cell efficiency for all tested ruthenium DSCs. While, as expected, the V_OC_ was not strongly altered by the introduction of AuNPs, it was found that the J_SC_ increased significantly by 4.67% (Ru N719), 9.29% (Ru N749) and 16.18% (Ru N3), leading to increased power efficiencies of 14.6%, 18.9%, and 25.5%, respectively. The plasmonic enhancement pro­duced by this method of efficiently incorpora­ting AuNPs was also characterized and confirmed by absorbance measurements. It was concluded that the used size distribution of AuNPs, immobilized via short dithiodibutyric acid linkers is responsible for the increase in performance, due to their high extinction, scattering properties and plasmonic enhancement features.

The presented systems also show long term stability as repeated tests after 3 months have established where the performance of the cells remained basically unaltered.

It is obvious that size distributions of plasmonically active metal NPs can be tailored to complement most dyes used in sensitized solar cells with the only limit regarding the shortest supportable wavelength because of the metal’s plasma frequency. However, changing the metal of the NPs. e.g. from Au to Ag or Al is moving the plasma frequency up and thus supports shorter wavelengths in DSC enhancement.

A future strategy is to use plasmonic enhancement not on dye systems which are already near optimal for harvesting across the entire visible range but to use the extra design freedom from plasmonic enhancement e.g. on sensitizer systems (dyes, quantum dots) which can still and highly efficiently inject electrons at NIR wavelengths but might have deficiencies across other parts of the spectrum. These deficiencies can then be removed by plasmonic enhancement and an overall increased efficiency of the total cell may be achieved.

## Supporting Information

Figure S1 **SEM analysis used to determine: (A) the overall morphology of the TiO_2_ nanostructured layer and the conjugation of optically dispersing TiO_2_ particles with the TiO_2_ nanoparticles scaffold; (B) the average diameter of the TiO_2_ nanoparticles: 20 nm; (C) the diameter distribution of the optically dispersing** ***TiO_2_ particles:*** **120–190 nm.**
(TIFF)
