# Supplementary figures and images for: Plasmonic Enhancement of Dye Sensitized Solar Cells via a Tailored Size-Distribution of Chemically Functionalized Gold Nanoparticles
Source: PLoS One. 2014 Oct 29;9(10):e109836. doi: 10.1371/journal.pone.0109836 (PMC4212914; doi:10.1371/journal.pone.0109836)

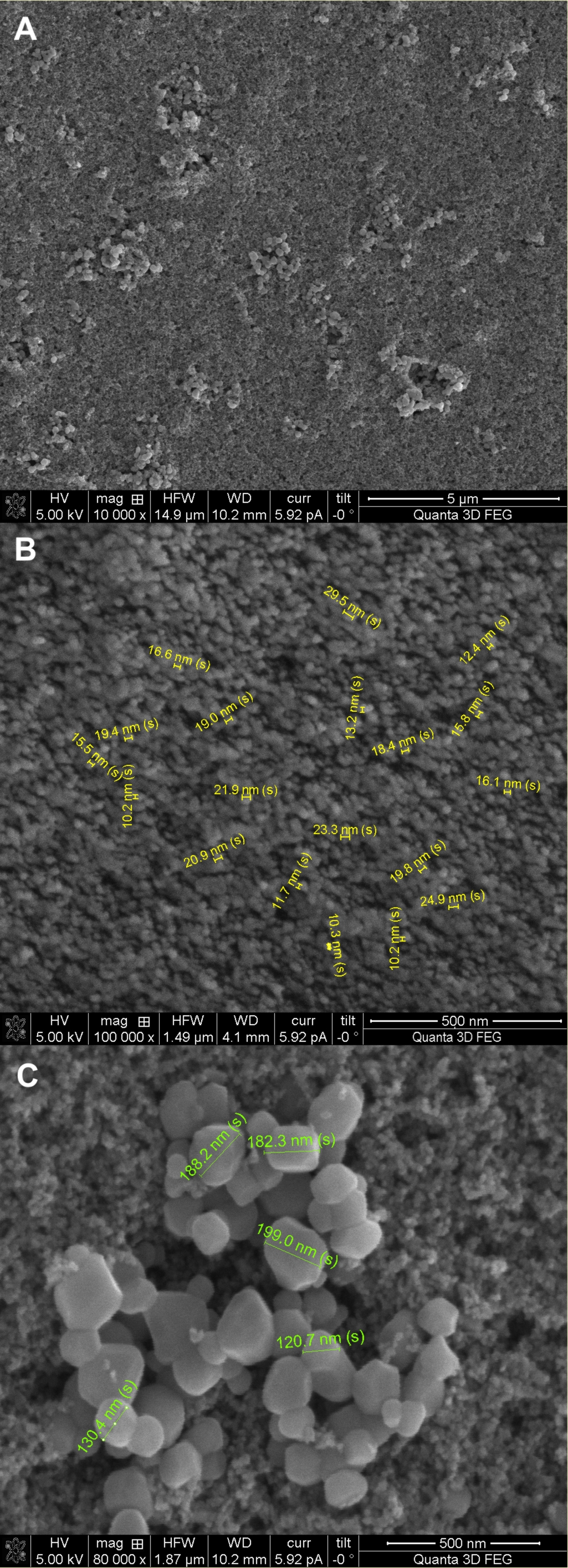

Supplement: Figure S1 — SEM analysis used to determine: (A) the overall morphology of the TiO2 nanostructured layer and the conjugation of optically dispersing TiO2 particles with the TiO2 nanoparticles scaffold; (B) the average diameter of the TiO2 nanoparticles: 20 nm; (C) the diameter distribution of the optically dispersing TiO2 particles: 120–190 nm. (TIFF) [file pone.0109836.s001.tiff]
